# Interprofessional Survey on Knowledge and Attitudes on Oral Health among Nurses in France

**DOI:** 10.3290/j.ohpd.b4586807

**Published:** 2023-11-02

**Authors:** Abid Bossouf, Céline Sabourin, Carina Pop, Nicolas Giraudeau, Camille Inquimbert

**Affiliations:** a Dentist, Department of Public Health, Faculty of Dental Medicine, University of Montpellier, Montpellier, France. Collected the data, participated in the initial data analysis, reviewed the final draft for academic content, approved the final manuscript.; b PhD Student, Department of Public Health, Faculty of Dental Medicine, University of Montpellier, Montpellier, France. Collected the data, participated in the initial data analysis, approved the final manuscript.; c Dentist, Department of Public Health, Faculty of Dental Medicine, University of Montpellier, Montpellier, France. Collected the data, participated in the initial data analysis, wrote the manuscript, approved the final manuscript.; d Dentist, Department of Public Health, Faculty of Dental Medicine, University of Montpellier, Montpellier, France. Wrote the manuscript, approved the final manuscript.; e Dentist, Department of Public Health, Faculty of Dental Medicine, University of Montpellier, Montpellier, France. Designed the data tool, wrote the manuscript, analysed the data, critically reviewed and approved the final manuscript.

**Keywords:** attitude, knowledge, literacy, nurses, oral health prevention, training

## Abstract

**Purpose:**

Healthcare professionals (HCPs) play a key role in improving the health literacy of patients. Since oral health is an essential part of overall health, the objective of this study was to assess the knowledge and attitudes about oral health among registered nurses.

**Materials and Methods:**

A four-component questionnaire was used to assess the oral health training, oral health knowledge and attitudes of registered nurses. Participants were recruited from the city of Montpellier, France, and the surrounding area between May and June 2022 via e-mail and social media.

**Results:**

In total, 416 responses were included in our study. Only 35.8% of nurses reported that they had received specific training on oral health and 24.3% had never advised patients to consult a dentist. Participants demonstrated good overall knowledge, but stated there were weaknesses in a variety of areas, such as oral health in children. The nurses proposed methods to improve understanding of the importance of oral health, namely by setting up training courses and better interdisciplinary collaboration.

**Conclusion:**

Our study showed that some aspects of oral health are not well understood by nurses. Initial training should be improved and supplemental training should be offered to improve the knowledge, attitude and practices of nurses in order to improve patient care.

Oral health is an essential part of the psychosocial well-being and general health of the individual.^16^ The World Health Organisation defines oral health as “the state of the mouth, teeth and orofacial structures that enables individuals to perform essential functions, such as eating, breathing and speaking, and encompasses psychosocial dimensions, such as self-confidence, well-being and the ability to socialise and work without pain, discomfort and embarrassment” and that it “varies over the life course from early life to old age, is integral to general health and supports individuals in participating in society and achieving their potential.”^37^

The high prevalence of oral diseases is a public health concern. Worldwide, 3.5 billion people are affected by oral diseases and over 514 million children suffer from caries in primary teeth. In France, in 2019, almost 30% of children between 1 and 9 years old had untreated caries in their deciduous teeth.^38^ However, most oral health problems are avoidable if treated at an early stage, with prevention being key in the fight against oral disease.

Nurses may have a role to play in providing oral health information, since they are particularly well placed to communicate with various healthcare professionals (HCPs) in their professional activity and also have a close interaction with patients, whether in the hospital, clinical or home settings.^1,11,18,26^ Nurses may be important in detecting oral diseases and educating patients.^19,28^

The literature has shown that oral health education can have a positive effect on the knowledge, attitude and practices of nurses.^13,22,27^ However, studies showed that nurses had incomplete knowledge on oral health.^17,25,33,40,41^

KAP (knowledge, attitudes, practices) surveys identify gaps in knowledge, cultural beliefs or behavioural patterns. The KAP survey is based on items formulated in questionnaires that can provide both quantitative and qualitative information. The aim of such a survey is to gather information about a specific target group. This is because the oral health KAP of healthcare professionals have a major influence on the community, as they can provide oral health education from the very first contact with patients.

The main objective of this study was to assess the oral health knowledge, attitude and practices among French nurses in the city of Montpellier and the surrounding area. The secondary objective was to gather nurses’ opinions on the types of prevention support that needed implementation after the study.

## Materials and Methods

### Sample and Setting

The questionnaire was created using Google Forms and participants were recruited by professional organisations (Council of the Order, Regional Association of Health Professionals), through private groups on social media sites, and via e-mail between May and June 2022, in the city of Montpellier and the surrounding area (France). A simple random sampling technique was used. 12,456 certified, registered nurses were targeted for the study. The minimum sample size was calculated to be 373 subjects, by assuming a 5% level of significance and 95% confidence interval. Nurses who had graduated from nursing school outside France or who were students at the time of the study were not included.

The Montpellier University Hospital Institutional Review Board (IRB) granted permission for the use of the collected data under the acceptance number 2022_10_202201250.

### Survey Design

An observational, descriptive, cross-sectional, multi-center study was conducted using a self-administered questionnaire. This study was part of a larger project called “Montpellier Santé Orale,” and similar studies have been conducted for midwives, speech therapists, physiotherapists, pharmacists, pediatricians and medical general practitioners (GPs).

### Recruitement and Data Collection

The questionnaire was developed as part of a collaborative multi-disciplinary project by the authors after reviewing previous studies and contextualised to the study area.^3,4,5,17^

The questionnaire integrated both quantitative and qualitative elements (see Supplementary Material 1). It was divided into four sections. There were three question types: five open-ended questions, eight yes-or-no questions and five multiple-choice questions. The first section collected information about the survey respondents’ background and training. The second section (14 questions) assessed their understanding of the relationship between oral health and general health (theme 1). The third section (16 questions) assessed the respondents’ understanding of prevention, hygiene habits, and the role of food/drink in oral health (theme 2). Finally, the fourth section gathered information on communication preferences for oral health prevention among nurses, but also for the general population and a part for free-text response.

### Data Analysis

Incomplete questionnaires were excluded from the study. Microsoft Excel and Stata software Version 16.1. (Stata; College Station, TX, USA) was used to perform the statistical analysis, and the results were presented in percentages (%). Participants with missing data were excluded from any analyses involving that data. Data regarding the oral health literacy of midwives was summarised using descriptive statistics including frequencies, means and standard deviations. The Mann-Whitney U-test was used to check whether there was a difference in correct responses, depending on the initial training. The relationships between the quantitative variables were analyzed using the χ^ 2^ test. The significance threshold was at 5%.

## Results

### Participants (Table 1)

**Table 1:** Characteristic of the participants

	n	%
**Gender**		
Male	59	14.2
Female	357	85.8
**Year of graduation**		
Before 2010	216	51.9
After 2010	200	48.1
**Age in years**		
< 30	88	21.2
31 – 40	133	32
41 – 50	116	27.9
51 – 60	67	16.1
> 60	12	2.9
**Work setting**		
Public hospital	263	63.2
Private practice	124	29.8
**Other**	29	7
Oral health education during initial training		
No	267	64.2
Yes	149	35.8
**Oral health education after initial training**		
No	260	62.5
Yes	156	37.5

We received 418 responses to the questionnaire in the two-month period (response rate: 3.35%). Two responses were excluded because the participants were not certified as registered nurses. In total, 416 nurses were included in the study. Most participants were female (n = 357, 85.8%) and were aged between 21 to 79 years (mean = 40.26; SD = 11.01). There was no statistically significant difference found in the sex of the respondents in relation to the age distribution (χ^2^ test, p = 0.43).

Most registered nurses completed their training (54.8%) in the city of Montpellier or the surrounding area. More than half of the registered nurses (n = 216, 51.9%) were accredited between 1971 and 2009. Most participants practiced in a public hospital (63.2%), followed by private practice (29.8%). Twenty-nine participants (7%) responded “other”. These participants worked in a variety of different settings, including a residential care home, neurology network, cruise company, nursery, dialysis center, infection control center, assisted living facility, charity, laboratory, and health network.

Only 35.8% of participants had received oral health education during their initial training and 5.5% had received specialised training on oral health since receiving their certification.

### Knowledge

To the questions on the relationship between oral health and general health (theme 1), we received correct answers 69.2% of the time (Table 2). The questions where participants answered least correctly were about respiratory problems and the position of the tongue in child facial growth, the bidirectional correlation between periodontal disease and diabetes control. Furthermore, knowledge about preventive dental examinations during pregnancy and that oral bacteria can reach the amniotic fluid and lead to infections in pregnant women was often scant. The most often correctly answered question was about tobacco use and that it increases the risk of upper respiratory tract cancer (96.8% of participants answered this correctly).

**Table 2:** Knowledge of nurses on the relationship between oral health and general health in the Occitanie region of France (Theme 1)

Statement	True/False	Correct responses	
		n	%
Studies show that there is little correlation between oral health and general health.	False	386	92.2
Oral bacteria can spread through the organism via the blood and airways.	True	379	91.1
Oral bacteria can reach the amniotic fluid and lead to infections in pregnant women.	True	225	54.1
There is a bidirectional correlation between periodontal disease and diabetic control.	True	212	50.9
Tobacco use increases the risk of upper respiratory tract cancer.	True	403	96.8
There is an oral prevention examination 100% covered by Health Insurance from the 4th month of pregnancy.	True	214	51.4
Dental care during pregnancy is limited to emergency care.	False	372	89.4
The M’T Dents (prevention examination) concerns children from the age of 3.	True	297	71.4
Oral bacteria can increase the risk of cardiovascular disease.	True	333	80.1
Heredity can play a role in certain pathologies, but this is not oral pathologies.	False	368	88.5
The premature loss of baby teeth has no effect on permanent dentition.	False	344	82.7
Respiratory problems in children can affect facial growth.	True	138	33.2
The position of the tongue is an essential growth factor in skull development.	True	216	51.9
Good oral health reduces the risk of lung disease.	True	260	63.5
Total correct responses			69.2

Regarding prevention, hygiene habits, and the role of food/drink in oral health (Table 3) (theme 2), we received correct responses 73.3% of the time. The least-often correctly answered questions were about the type of toothbrush: the number of bristles on a toothbrush influences the quality of brushing. Only half of the nurses knew what fluoride and it is recommended before the age of 6. Participants demonstrated relatively poor knowledge of good oral hygiene in children. Although most participants were aware that snacking increases the likelihood of cavities and that acidic drinks lead to dental erosion, we observed a lack of knowledge concerning the risk of cavities (early childhood caries) in very young children, in the case of prolonged use of a feeding bottle during the day). Toothbrushing was reported as being necessary when the first primary teeth appear; however, few nurses were aware of the necessity to continue brushing the oral mucosa even in the absence of teeth, and the need to clean removable orthodontic appliances with a specific brush.

**Table 3:** Knowledge of nurses regarding prevention, hygiene habits, and the role of food/drink in oral health in the Occitanie region of France (Theme 2)

Statement	True/False	Correct responses	
		n	%
Brushing your teeth in the morning and at noon is equivalent to brushing your teeth in the morning and evening.	False	408	98.1
An electric toothbrush is always more effective than a manual toothbrush, even if you use a good brushing technique.	False	190	45.7
Toothbrushes with soft, medium or hard bristles are equally effective as long as the correct brushing technique is used.	False	197	47.4
The number of bristles on a toothbrush influences the quality of brushing.	True	170	40.0
A toothbrush with hard bristles can cause irreversible damage to the gum tissues as well as to the dental tissues.	True	314	75.5
It is necessary to clean the interdental spaces with dental floss or interdental brushes daily in addition to the brushing.	True	331	79.6
Mouth cleaning is no longer useful when you have no more teeth.	False	409	98.3
Fluoride is a natural mineral that can be found in tap water, tea or some fish.	True	216	51.9
For children under 6, toothpaste should not be fluoridated.	False	191	45.9
Drinking sodas too often causes dental erosion.	True	388	93.3
The products used by the dentist for dental whitening are similar to those found over the counter.	False	414	99.5
The cleaning of dental prostheses (removable) can be done with a manual or electric toothbrush.	False	204	49.0
It is not necessary to clean baby teeth as soon as they appear.	False	399	95.9
Intake of sugary/acidic foods or drinks throughout the day increases the risk of developing caries.	True	394	94.7
A 4-year-old child can have cavities because of drinking milk from a bottle (prolonged use of the bottle during the day or when falling asleep).	True	284	68.3
Risk of caries decreases when the quantity of saliva decreases.	False	369	88.7
Total correct responses			73.3

We found no statistically significant difference in the number of correct responses regarding the place of work, nor according to whether or not the participant had received specific training during their qualification (Mann-Whitney U-test, p = 0.08 for theme 1, p = 0.32 for theme 2).

We found no association between the year of the nursing certification and the rate of correct responses (Mann-Whitney U-test, p = 0.58 for theme 1 and p = 0.49 for theme 2).

### Attitude and Practice

In our study population, 37.5% of respondents reported that they regularly advised patients to consult a dentist, 38.7% did so occasionally and 24.3% never advised patients to consult a dentist. Nurses in private practice, clinics as well as those in “other” structures were statistically significantly more likely to advise patients to consult a dentist than those working in public hospitals (χ^2^ test, p = 0.0001).

### Communication Preferences for Oral Health Prevention

**Fig 1:**
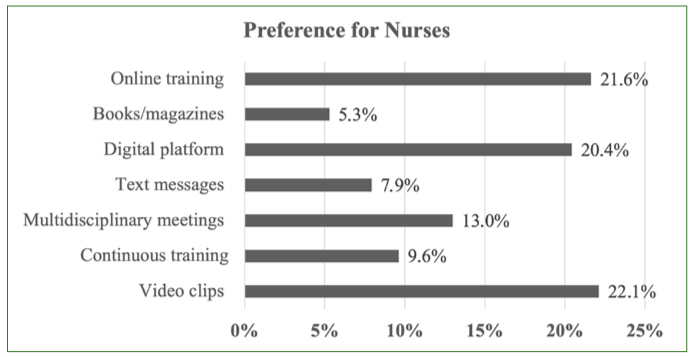
Communication preferences for oral health prevention for nurses.

**Fig 2:**
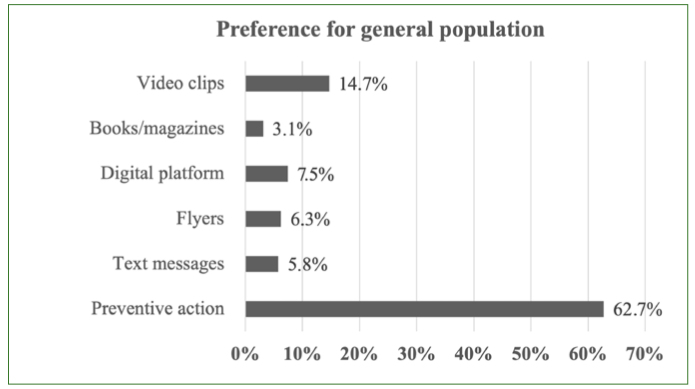
Communication preferences for oral health prevention for general population.

The most popular methods for receiving training/information about oral health among nurses (Fig 1) were all based on digital communications such as video clips, online training and digital platforms. Multidisciplinary evening events, an oral-health diploma, SMS messages and books/magazines were less popular (Fig 2). The opposite trend was observed for general population outreach methods (Fig 2). The most common first choice was actions in the field (62.7% of responses).

### Nurses’ Perceptions

In the open-ended part of the questionnaire, participants commented on possible improvements in the training of nurses, the management of patients in hospital, the role of dentists in improving patient care and how to raise awareness in the general population. Participants mentioned a general lack of knowledge about oral health and felt that the subject had not been covered well in their training. Eighty-two (19.7%) participants expressed a desire to receive more training. The following comments were given:

- “There is a need to standardise oral health education across training centers.”
- “We should consider providing information using posters in treatment rooms.”
- “Training courses as part of our initial training or continuing professional development should be created leading to a diploma in oral health.”

Many comments referred to the poor oral health of at-risk patients such as those being treated in psychiatry, intensive care, geriatrics, ENT or in oncology. Participants suggested the following to address this:

- Preventive actions such as reminding people of the importance of oral hygiene and screening during consultations.
- Having a designated person responsible for dental hygiene in at-risk departments.
- Development of teleconsultations for oral health in hospital wards.

## Discussion

This is the first study conducted in France to assess the knowledge of oral health among nurses. Our pilot study may add valuable insight about how to improve oral health in patients. The anonymous nature of our study allowed participants to respond candidly, and the use of digital platforms to recruit participants allowed us to gather perspectives from across different workplaces. Overall, the responses to our questionnaire were thorough and we were able to include all the responses into our analysis.

The ratio of male to female participants and the mean age in our study was representative of the general population of nurses in France, which is a female-dominated profession with 86.6% of nurses being women in 2021, and the mean age in 2022 was 40.8 years (in our study 40.26 years).^7^

Regarding the type of practice of the respondents, the 2021 nursing statistics conveyed that 64% of nurses practiced in public hospitals.^7^ Our study had similar findings for the public hospital nurses.

Our study showed that only 35.8% of the participants had received specialised training in oral health during their initial training. We found no statistically significant difference in knowledge according to the year of certification. This may suggest that the 2009 national teaching reforms did not improve oral health education in this profession.^23^ We also found no statistically significant difference in knowledge among nurses who had received training or did not. A study in Ethiopia found that nurses with a diploma or higher were five times more likely to have good knowledge than nurses with a degree (AOR = 5.05, 95% CI: 1.85–13.83).^4^ This may suggest that the training provided in France has thus far not been adequate, and may also highlight the need for ongoing training as part of a nursing continuing professional development. In line with other studies, there is a need for better training.^5,8,10,31^

Approximately one-quarter of the nurses we surveyed responded that they never advise patients to consult a dentist. This is often due to a lack of time or a lack of referring dentists.^24,36^ Additionally, we found that hospital nurses were less likely to refer a patient to a dentist than nurses in other settings. This may be because a hospital setting is less conducive to this kind of referral, since pathologies treated in hospitals are often more severe, so oral health seems less of a priority.^9,30,35^

In general, participants had a good understanding of the relationship between oral bacteria and bacteria in the rest of the body. They were also aware of the direct link to cardiovascular disease. However, they were often unaware of the link between poor oral health and pathologies such as diabetes and lung disease, although nurses they play an essential role in preventing the complications of these diseases.^2^ There was a particular lack of knowledge concerning oral health during pregnancy and the associated risks to the mother and her unborn baby. A 2016 study recommends developing an oral-health education programme to improve nurses’ knowledge of and attitudes to oral health during pregnancy and oral health care for pregnant women.^29^ In addition, they did not know that the health insurance system provides a free preventative dental consultation during pregnancy.

We observed a lack of understanding about fluoride and its importance in preventing cavities. This may be due to the current trends towards toothpaste without fluoride (replacing it with essential oils or other natural-based products) that is increasingly observed on the internet. This lack of knowledge has been highlighted in several studies.^3,6^ We know that the level of knowledge about oral health is linked to oral health behaviour, which includes brushing time, use of fluoride toothpaste and regular visits to the dentist.^3^ However, fluoride has irrefutably been proven to be the most effective anti-caries agent, and any intoxication risks are due to egregious misuse.^32^ It may be therefore particularly necessary for both nurses and patients to be educated on this subject to combat misinformation.

The poor knowledge of good oral hygiene in children observed in our study may need to be a focal point of future training as well as information campaigns.

The free-text responses revealed the need to improve initial and continued training about oral health among nurses. Participants suggested a range of ways to implement this, including training courses in hospitals, a university diploma, and informative posters. Participants also suggested having a designated person responsible for oral health available in at-risk wards and developing teleconsultations in light of the general lack of availability of dentists in hospitals. Evidence is now emerging which supports the efficacy of teledentistry. The use of e-health and e-oral health technologies enables effective remote screening, diagnosis, faster referral from primary care to specialist services, reduced amount of travel to urban sites, and increased cost-effectiveness of health care.^12,14,15^

Multiple comments referred to the poor oral health of patients with psychiatric disorders. A psychiatric patient’s lack of cooperation or resistance to treatment as well as a healthcare worker’s misinterpretation of a patient’s physical reactions can be barriers to providing dental care. One solution could be to create tailored protocols to help healthcare teams improve the day-to-day care of this population, as suggested in a study conducted by Gers University Hospital, France.^21^ It may be helpful to set up collaborative projects between dentists and nurses, which has been shown to improve confidence levels among nurses and thus the referral rates to dentists from nurses, as has been done in the USA.^20^ These collaborations could improve the knowledge and confidence in prevention and screening among nurses.^34^

Our results also revealed that nurses would be most interested in receiving information/training through digital resources such as short videos, online training and digital platforms. This preference should be taken into consideration when developing additional resources. The WHO launched the mOralHealth Programme and the Handbook on “Mobile technologies for oral health: An implementation guide” in 2021.^39^ The programme proposes a specific module on mOralHealth training to improve knowledge and skills of frontline health workers on oral health, with the use of digital technology to reach healthcare professionals.

Regarding this study’s limitations, respondents worked in the city of Montpellier and the surrounding area, and all the hospital nurses worked in the city of Montpellier, so that the generalisability of this study may be restricted. Additionally, the format of the questionnaire meant that the results had to be extracted manually, which may have led to errors. Because the questionnaire was voluntary, a selection bias may have resulted. This may mean that our participants had greater knowledge than that found in the general nursing population and that they may have had higher interest in further training. Lastly, since the questionnaire was online, we cannot guarantee that participants only used their own knowledge to answer the questions. The percentage of correct responses may therefore be higher in our sample than in reality.

## Conclusions

Improvement of oral health literacy among nurses could positively impact patients, who could then take a more active role in their own health. Initial training including a module dedicated to oral health that is specifically focused on prevention methods and tailored to a patient’s age, as well as improving the training available through continuing professional development programmes may be necessary in the future.

## Supplementary material

**Table:** Questionnaire for the assessing the oral health training of registered nurses in France

Questionnaire: Nurses’ knowledge of oral health
*The WHO definition of oral health is as follows:* *“Oral health is the state of the mouth, teeth and orofacial structures that enables individuals to perform essential functions, such as eating, breathing and speaking, and encompasses psychosocial dimensions, such as self-confidence, well-being and the ability to socialize and work without pain, discomfort and embarrassment. Oral health varies over the life course from early life to old age, is integral to general health and supports individuals in participating in society and achieving their potential.”* *The aim of this study is to assess knowledge of oral health among nurses.*
Question 1: What is your sex? Male Female
Question 2: How old are you? ____________
Question 3: Where did you complete your certification? (postcode) _____________
Question 4: In what year did you receive your certification? _____________
Question 5: Where are you currently practicing? (postcode) _____________
Question 6: In what setting do you currently work? Hospital Clinic Private practice Other: _____________
Question 7: Did you receive specific training in oral health during your training? Yes No
Question 8: If yes, do you believe it was sufficient? Yes No
Question 9: Have you received any additional training on oral health since you qualified? Yes No
Question 10: Of the following statements related to oral/general health, please select those you agree with: Studies show that there is little correlation between oral health and general health Oral bacteria can spread through the organism via the blood and airways Oral bacteria can reach the amniotic fluid and lead to infections in pregnant women There is a bidirectional correlation between periodontal disease and diabetic stability Smoking increases the risk of cancer in the upper respiratory tract The French medical insurance system fully refunds a dental consultation from the 4th month of pregnancy Dental care during pregnancy is limited to emergency care The M’T dents programme (oral health prevention) is available to children from 3 years old. Oral bacteria can increase the risk of cardiovascular disease Genetics can play a role in some pathologies, but not in oral pathologies Early loss of milk/baby teeth has no effect on adult teeth. Respiratory problems in children can have an effect on facial development. Tongue position is a key factor in skull development. Good oral health reduces the risk of lung disease
Question 11: Of the following statements related to prevention and food/drink, please select those you agree with: Brushing one’s teeth in the morning and at midday is the same as brushing one’s teeth in the morning and the evening. An electric toothbrush is always more effective than a manual toothbrush, even with a good toothbrushing technique. Toothbrushes with soft, medium or hard bristles are equally effective if used with a good toothbrushing technique. The number of bristles on a toothbrush affects the quality of brushing. A hard-bristled toothbrush can cause irreversible damage to gum and tooth tissue. It is essential to clean the interdental spaces daily with floss or interdental brushes in addition to toothbrushing. Toothbrushing/cleaning the mouth is no longer useful when the person has no teeth left. Fluoride is a natural mineral found in tap water, tea, and some fish. Children under 6 years old should use fluoride-free toothpaste. Drinking carbonated drinks too often causes tooth erosion. Products used by dentists for teeth whitening are similar to those available off the shelf. Removable dental appliances are cleaned using either a manual or electric toothbrush. It is not necessary to brush milk/baby teeth as soon as they appear. Eating/drinking sugary or acidic food/drink throughout the day increases the risk of developing cavities. A 4-year-old child can acquire cavities from drinking milk from a bottle (prolonged use of a bottle during the day or while going to sleep) The risk of cavities decreases as the amount of saliva decreases.
Question 12: Do you advise patients to consult a dentist, or do you speak to the patient’s general practitioner? Yes, regularly Yes, occasionally No, never
Question 13: What kind of intervention do you think would be helpful for you as a healthcare professional? (by order of preference) Video clips Continuous training Multidisciplinary meetings Text messages Digital platform Books/magazines Online training
Question 14: What kind of intervention do you think would be helpful for the general population? (by order of preference) Preventive action Text messages Flyers Digital platform Books/magazines Video clips
Question 15: Do you have any comments/suggestions/ideas?
*Thank you for your participation*
